# Self-formed compositional superlattices triggered by cation orderings in *m*-plane Al_1−*x*_In_*x*_N on GaN

**DOI:** 10.1038/s41598-020-75380-3

**Published:** 2020-10-29

**Authors:** Shigefusa F. Chichibu, Kohei Shima, Kazunobu Kojima, Yoshihiro Kangawa

**Affiliations:** 1 Institute of Multidisciplinary Research for Advanced Materials, Tohoku University, 2-1-1 Katahira, Aoba, Sendai 980-8577 Japan; 2 Research Institute for Applied Mechanics, Kyushu University, Kasuga, Fukuoka 816-8580 Japan

**Keywords:** Condensed-matter physics, Materials for optics

## Abstract

Immiscible semiconductors are of premier importance since the source of lighting has been replaced by white light-emitting-diodes (LEDs) composed of thermodynamically immiscible In_*x*_Ga_1−*x*_N blue LEDs and yellow phosphors. For realizing versatile deep-ultraviolet to near-infrared light-emitters, Al_1−*x*_In_*x*_N alloys are one of the desirable candidates. Here we exemplify the appearance and self-formation sequence of compositional superlattices in compressively strained *m*-plane Al_1−*x*_In_*x*_N films. On each terrace of atomically-flat *m*-plane GaN, In- and Al-species diffuse toward a monolayer (ML) step edge, and the first and second uppermost < $\stackrel{-}{1}\stackrel{-}{1}20$> cation-rows are preferentially occupied by Al and In atoms, respectively, because the configuration of one In-N and two Al-N bonds is more stable than that of one Al-N and two In-N bonds. Subsequent coverage by next < $\stackrel{-}{1}\stackrel{-}{1}20$> Al-row buries the < $\stackrel{-}{1}\stackrel{-}{1}20$> In-row, producing nearly Al_0.5_In_0.5_N cation-stripe ordering along [0001]-axis on GaN. At the second Al_0.72_In_0.28_N layer, this ordinality suddenly lessens but In-rich and In-poor < $\stackrel{-}{1}\stackrel{-}{1}20$>-rows are alternately formed, which grow into respective {0001}-planes. Simultaneously, approximately 5-nm-period Al_0.70_In_0.30_N/Al_0.74_In_0.26_N ordering is formed to mitigate the lattice mismatch along [0001], which grow into approximately 5-nm-period Al_0.70_In_0.30_N/Al_0.74_In_0.26_N {$10\stackrel{-}{1}2$} superlattices as step-flow growth progresses. Spatially resolved cathodoluminescence spectra identify the emissions from particular structures.

## Introduction

Incandescent bulbs and fluorescent tubes are progressively replaced by compact and high-efficiency white light-emitting-diodes (LEDs)^1^ composed of In_*x*_Ga_1-*x*_N quantum-well (QW) blue LEDs^2^ and yellow phosphors, thanks to the threading dislocation (TD)-tolerant radiation probability^3–5^ of localized excitons in low InN mole fraction (*x*) In_*x*_Ga_1-*x*_N alloys. Consequently the research fields of thermodynamically immiscible III-nitride alloys^6–10^ have been exciting more than 25 years. For realizing high-efficiency light sources for high color-rendering-index lighting, curing, skin therapy, and sterilization/disinfection, near- to far-ultraviolet light-emitters of similarly high radiative efficiency as In_*x*_Ga_1−*x*_N are indispensable. Especially, deep ultraviolet (DUV) LEDs are a key device for biochemical detection, non-line-of-sight communications, and disinfection of viruses.

For the urgent development of DUV LEDs, Al_*x*_Ga_1−*x*_N alloys have been studied and great strides have been achieved^11–19^. Beyond Al_*x*_Ga_1−*x*_N, the use of immiscible^7,10,20–22^ Al_1−*x*_In_*x*_N alloys is an exotic way for realizing light-emitters^23,24^ operating in DUV to infrared wavelengths, because their bandgap energies (*E*_g_) cover from DUV (*E*_g_ = 6.01 eV for AlN) to infrared (*E*_g_ = 0.65 eV for InN) wavelengths. However, *c*-plane Al_0.82_In_0.18_N epilayers lattice-matched to GaN have been exclusively investigated as auxiliary components such as Al_0.82_In_0.18_N/GaN distributed Bragg reflectors^25^ and an Al_0.82_In_0.18_N barrier for GaN heterostructure field-effect transistors^26^. The major reason why Al_1−*x*_In_*x*_N have scarcely been investigated as light-emitting media includes the difficulties in growing Al_1−*x*_In_*x*_N containing low concentration of nonradiative recombination centers (NRCs), *N*_NRC_, as follows. Because AlN and InN have large lattice mismatch (Δ*a*/*a*) of approximately 14%, the delta-lattice-parameter (DLP) model^27^ predicts nonmiscible of Al_1-*x*_In_*x*_N alloys^7,10,20–22^. Also, because optimum film growth temperatures (*T*_g_) for metalorganic vapor phase epitaxy (MOVPE) of AlN (> 1600 °C) and InN (< 400 °C) are significantly different, Al_1-*x*_In_*x*_N alloys suffer from kinetic phase separation due to instantaneous evaporation of In or InN from the surface^7^. Consequently, there have been few reported results on a near-band-edge (NBE) emission of Al_1−*x*_In_*x*_N films^5,23,28–34^ and QWs^24,35^, and the NBE emission exhibited large full-width at half-maximum (FWHM) value of a few hundred meV^5,23,24,28–35^ and Stokes’ shifts (SSs) larger than several hundred meV or nearly 1 eV, which is the energy difference between the absorption and emission. From a different perspective, large SS^29,30,32,34^, weak thermal quenching^23,32^, and morphology-insensitive cathodoluminescence (CL) intensity mapping images^23^ for the NBE emission owing to short minority-carrier (hole) diffusion length (*L*_p_) likely suggest that the NBE emission originates from certain localized states. Such localized states may have advantages^3–5^ in obtaining high quantum efficiencies (QE). However, insufficient photoluminescence (PL) lifetimes (${\tau }_{\mathrm{PL}}$) at room temperature, which nearly represents the nonradiative lifetime (${\tau }_{\mathrm{NR}}$), shorter than 80 ps^23,29,33^ indicates the difficulties in decreasing *N*_NRC_. Moreover, there have been limited publications^23,31,32,34^ on an NBE emission of nonpolar-plane Al_1-*x*_In_*x*_N, although (Al,In,Ga)N grown in off-polar orientations^36,37^ are a promising candidate for opto- and electronic devices of ultimate performance: as summarized in Refs.^5,23,36,37^, nonpolar planes are electrically-neutral and polarization discontinuity normal to heterointerfaces does not exist since the *c*-axis is parallel to the interface. Such QWs do not suffer the deleterious polarization-induced electric field^5,23,36–38^ and therefore regular conduction- and valence-band profiles result in well-overlapped electron–hole wavefunctions^5,23,36–39^.

**Figure 1:**
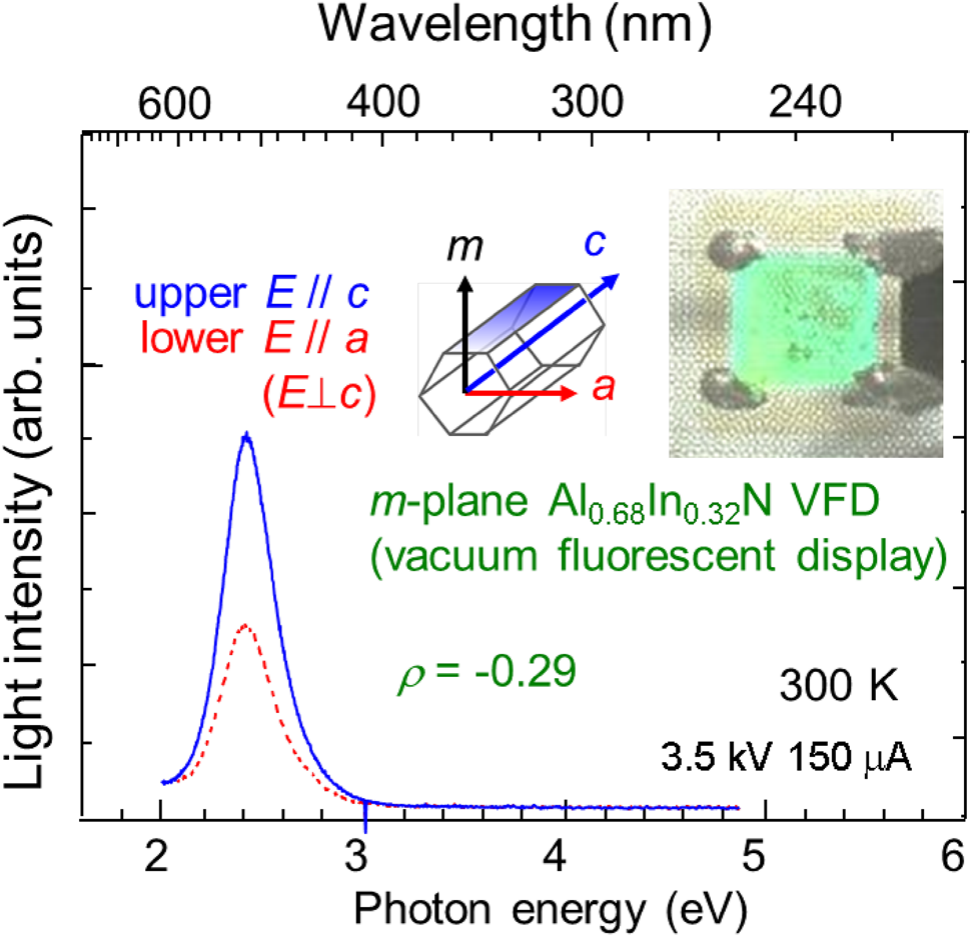
Polarized emission spectra of the green-light-emitting VFD device comprising of *m*-plane Al_0.68_In_0.32_N epitaxial nanoboards operated at 300 K. Although the magnitude of polarization (*ρ*) was not unity, the emission is essentially *E*//*c* polarized. Insets show the configuration of crystal axes and a photograph of the device under operation, taken from the surface through the grid electrodes: the shadows of the grids are superimposed on the emitting patterns. Partially reproduced with permission from Chichibu et al.^23^.

The authors grew *m*-plane Al_1−*x*_In_*x*_N on *m*-plane freestanding (FS)-GaN substrates by MOVPE^32^, which exhibited predominant NBE emission bands ranging from 5.81 to 2.39 eV with increasing *x*. By installing these films, planar vacuum fluorescent display (VFD) devices emitting polarized UV-C, blue, and green lights were trial-manufactured^23^. In Fig. 1, polarized emission spectra of our Al_0.68_In_0.32_N green VFD device at 300 K are shown^23^. The insets show the configuration of crystal axes and a photograph of the device under operation. Although the magnitude of polarization ($\rho$) was not unity, the electric field (*E*) component of the light was polarized parallel to the *c*-axis (*E*//*c*). However, little is known about the luminescence mechanisms of Al_1−*x*_In_*x*_N alloys^5,23,24,28–35^ and it is worth clarifying the reason why Al_1−*x*_In_*x*_N exhibits defect-tolerant radiation probability. In this article, two distinct length-scale cation orderings are exemplified to explain the appearance and self-formation sequence of compositional superlattices (CSLs) in *m*-plane Al_0.72_In_0.28_N films, in which excitons are confined in Al_0.70_In_0.30_N layers. Then, so-called composition-pulling^21,40^ phenomenon is described. Finally, cross-sectional spatially resolved cathodoluminescence (SRCL) spectra are correlated with these particular zones.

## Results: compositional superlattices (CSLs) in a pseudomorphic Al_0.72_In_0.28_N film

**Figure 2:**
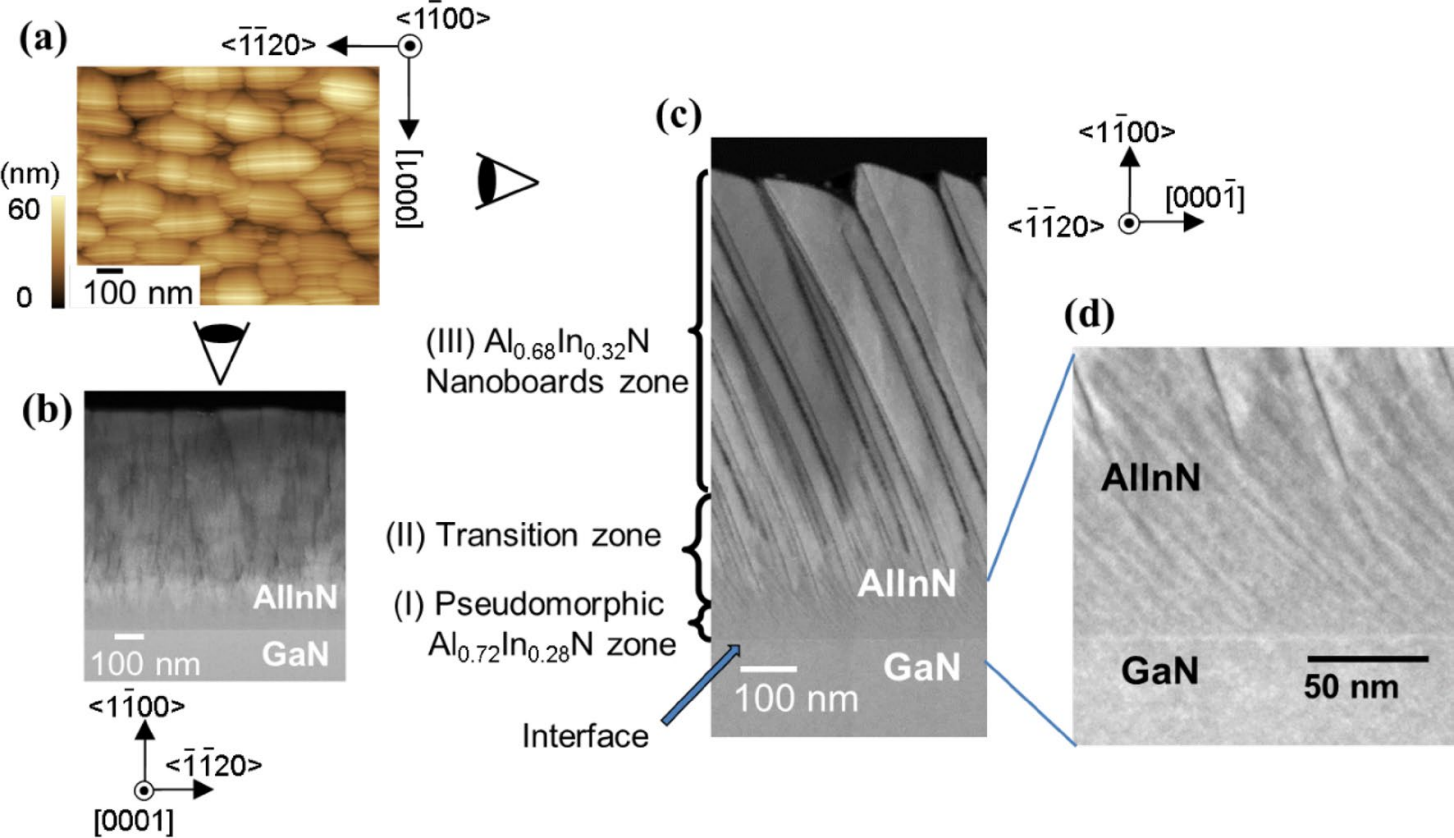
(**a**) Surface AFM image for the *m*-plane Al_0.7_In_0.3_N epitaxial structure grown on *m*-plane GaN substrate. Cross-sectional HAADF-STEM images taken from (**b**) (0001) and (**c**, **d**) {$11\stackrel{-}{2}0$} cross-sections. In (**a**), the directions of visions for (**b**) and (**c**, **d**) are schematically indicated by eyes beside the image. Panels (a), (c), and (d) are reproduced with permission from Chichibu et al.^23^.

**Figure 3:**
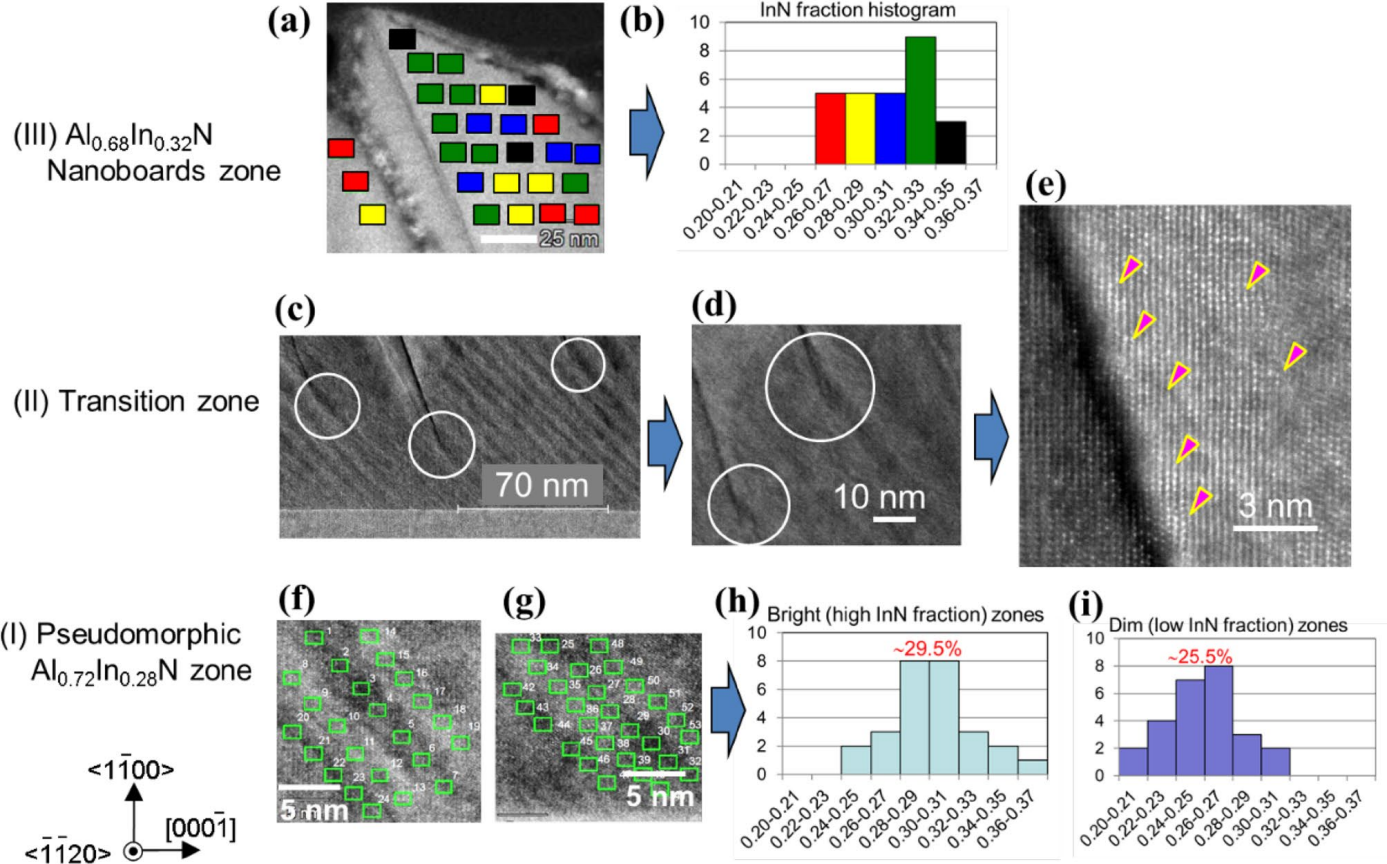
(**a**, **c**, **d**, **e**, **f**, **g**) Cross-sectional HAADF-STEM images for the *m*-plane Al_0.7_In_0.3_N epitaxial structure taken from {$11\stackrel{-}{2}0$} cross-section. In (**a**), exact locations where nanoprobe EDX composition analyses were carried out in “Nanoboards zone” are indicated, and corresponding local InN mole fractions *x* are shown using a frequency histogram in (**b**). In (**c**, **d**), the areas where the continuous film growths terminated in “Transition zone” are indicated by circles. In (**e**), high-resolution HAADF image taken around the starting area of the gap is shown. As indicated by triangles, slight changes in crystal orientations are observed. Local *x* values measured at the locations indicated by rectangles in (**f**) and (**g**) are summarized in frequency histograms shown in (**h**) and (**i**) for the bright (high *x*) and dim (low *x*) stripe-areas, respectively.

Figure 2 shows the structures of our *m*-plane Al_0.7_In_0.3_N epilayer^32^ used in the VFD device^23^ in Fig. 1. Its surface is composed of elliptic grains with the major axes almost parallel to < $\stackrel{-}{1}\stackrel{-}{1}20$ > *a*-axis, as shown in the surface atomic-force microscopy (AFM) image^23,32^ in Fig. 2a. For observing slight changes in crystallographic orientation and/or chemical composition, cross-sectional high-angle annular dark-field (HAADF) images were taken using a scanning transmission-electron microscopy (STEM). In Fig. 2a, the directions of visions for Fig. 2b from (0001) and Fig. 2c,d from {$11\stackrel{-}{2}0$} cross-sections are indicated schematically by eyes. These images (Fig. 2b,c) indicate that the epitaxial Al_1−*x*_In_*x*_N structure comprises of three zones, namely (I) approximately 60-nm-thick “Pseudomorphic zone”, (II) approximately 100 to 150-nm-thick nonuniform zone named “Transition zone”, and (III) approximately 500-nm-thick “Nanoboards zone”, as defined in Fig. 2c. The nanoboards are the elliptic grains seen in Fig. 2a. Corresponding magnified images are shown in Figs. 2d, 3f,g, Fig. 3c–e and a, respectively. As shown in Fig. 2c,d, Pseudomorphic zone is a three-dimensional (3D) film with regularly aligned stripes with bright and dim contrasts parallel to an {$10\stackrel{-}{1}2$} plane. The continuous film growth seems to terminate in Transition zone at the areas encircled in Fig. 3c,d, and a dense array of 80 to 100-nm-thick Al_1−*x*_In_*x*_N nanoboards^23^, which are divided by spatial gaps, align on {$10\stackrel{-}{1}3$} facets in Nanoboards zone, as shown in Fig. 2c. As similar samples^32^ have been characterized by using x-ray diffraction (XRD) measurements to have an epitaxial relationship with GaN underlayer, these Al_1−*x*_In_*x*_N structures are called as *epitaxial nanoboards*.

In order to quantify local *x*, nanometer-probe energy-dispersive x-ray (EDX) composition analyses were carried out. In Fig. 3a, exact locations where the nanoprobe EDX measurements were carried out are indicated by closed rectangles on the HAADF-STEM image. The frequencies of local *x* are shown using a histogram in Fig. 3b, where the colors of rectangles in Fig. 3a indicate the *x* values given in Fig. 3b. As shown, local *x* distributes from 0.26 ± 0.03 to 0.35 ± 0.03 with average *x* of about 0.31_5_. Although the error in *x* for the present EDX analysis is estimated to be ± 0.03, the finite extent in *x* being about 0.10 (0.26–0.36) is significant. In the bulk of nanoboards, remarkable accumulation of In is not found. These results mean that average *x* of the nanoboards are almost homogeneous but *x* has remarkable inhomogeneity with the length scale shorter than a few nm. This local inhomegeneity produces remarkable *E*_g_ inhomogeneity, because Al_1−*x*_In_*x*_N alloys of low to middle *x* have high derivative *dE*g(*x*)/*dx* value, which causes large alloy broadening^23,24,28–35^. In the present nanoboards, *E*_g_ for *x* = 0.26 and 0.35 are estimated according to Refs.^20,23,28–30,32,34,41–46^ to be approximately 3.42 and 2.75 eV, respectively, which account for SS of about 0.67 eV. This value reproduces the experimental result being 0.7 eV^23,32^. Therefore, such a few nm-scale inhomogeneity in *x* can explain the large SS and also large FWHM value of the NBE peak (approximately 0.3 eV in these Al_0.68_In_0.32_N nanoboards)^32^. We note that the VFD device shown in Fig. 1 is operated under the acceleration voltage (*V*_acc_) of 3.5 kV, and hence CL is generated in these nanoboards.

In contrast to Nanoboards zone, HAADF-STEM images of Pseudomorphic zone exhibit regularly aligned stripes with bright and dim contrasts parallel to {$10\stackrel{-}{1}2$} *r*-planes, as shown in Figs. 2c,d,3c,f,g. The magnified images of Fig. 2d are shown in Fig. 3f,g, in which exact locations for EDX measurements are indicated by open rectangles. In Fig. 3h,i, the frequencies of local *x* in the bright and dim stripes, respectively, in Fig. 3f,g are shown using histograms. As shown, the average *x* in the bright and dim stripes are obtained as 0.30 ± 0.03 and 0.26 ± 0.03, respectively, with finite extent of about 0.13. The result that brighter stripes in the HAADF images exhibit higher *x* is reasonable, because the brightness of HAADF images of a crystal structure is essentially proportional to square of the atomic number (*Z*). Although the estimated error value in *x* for the present EDX analysis is ± 0.03, Fig. 2c,d, 3c,f,g prove the presence of CSLs, and the accuracy of EDX seems sufficient. Accordingly, this Al_0.72_In_0.28_N pseudomorphic film is nothing but approximately 5-nm-period {$10\stackrel{-}{1}2$} Al_0.74_In_0.26_N/Al_0.70_In_0.30_N CSLs.

We note that *x* in the brighter zone within Transition zone in Fig. 2b,c is not higher than the other areas: i.e. the brightness change is likely due to slight changes in crystallographic orientations. In Fig. 3e, significant perturbations in lattice images are indicated by triangles. Also, the presence of a gap that is similar to deficiency of the material makes the images dim, as shown in Figs. 2c, 3c–e. Anyhow, the observed increase in average *x* from Al_0.72_In_0.28_N in Pseudomorphic zone to Al_0.68_In_0.32_N in partially strain-relaxed Nanoboards zone is known as the “composition-pulling effect”^21,40^ that stems from the presence of a lattice-mismatched substrate. The reason for the change in *x* under the same growth conditions includes the change in growing surface morphology triggered by lattice relaxation^21,40^, which takes place when the accumulated stress exceeds the elastic limit. In the present sample, the continuous film growth terminates at approximately 50–60 nm in Transition zone (Fig. 3c,d), where the spatial gaps are introduced instead of TDs.

## Discussion 1: cation-ordered Al_1−*x*_In_*x*_N on GaN and In-rich/In-poor *c*-planes in Al_0.72_In_0.28_N

**Figure 4:**
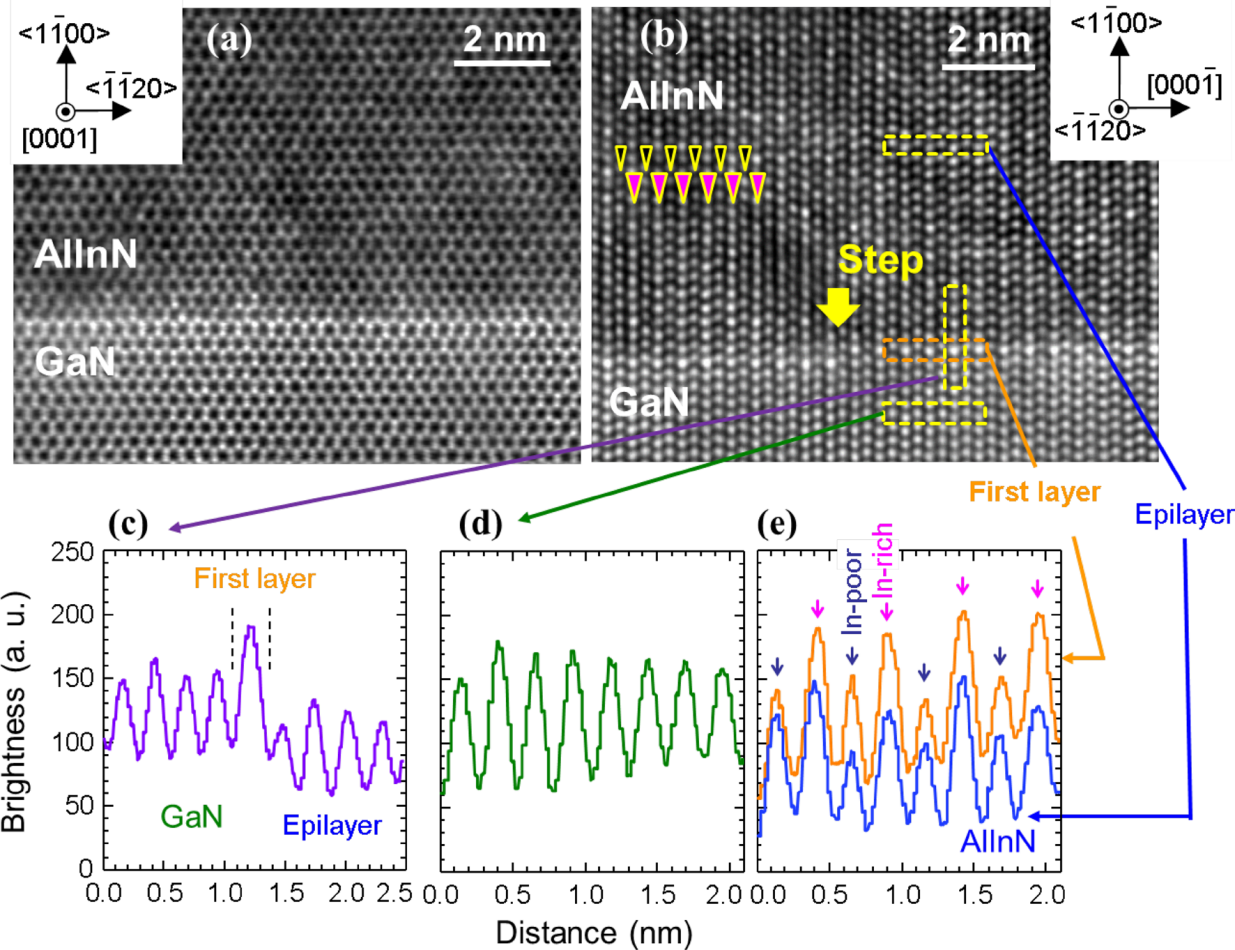
High-resolution cross-sectional HAADF-STEM images for the *m*-plane pseudomorphic Al_0.72_In_0.28_N/GaN heterointerface taken from (**a**) (0001) and (**b**) {$11\stackrel{-}{2}0$} cross-sections. In (**b**), In-rich (0001) plane (brighter row) and In-poor (0001) plane (dimmer row) are indicated by magenta and dark blue triangles, respectively. The location of a ML atomic step is indicated by the arrow. In (**c**–**e**), brightness profiles for the HAADF image enclosed by rectangles in (**b**) are shown. (**c**) A vertical brightness profile across the interface. (**d**) A lateral brightness profile for underlaying GaN. (**e**) Lateral profiles at the first layer (top trace) and in the bulk of Al_1−*x*_In_*x*_N (bottom trace).

**Figure 5:**
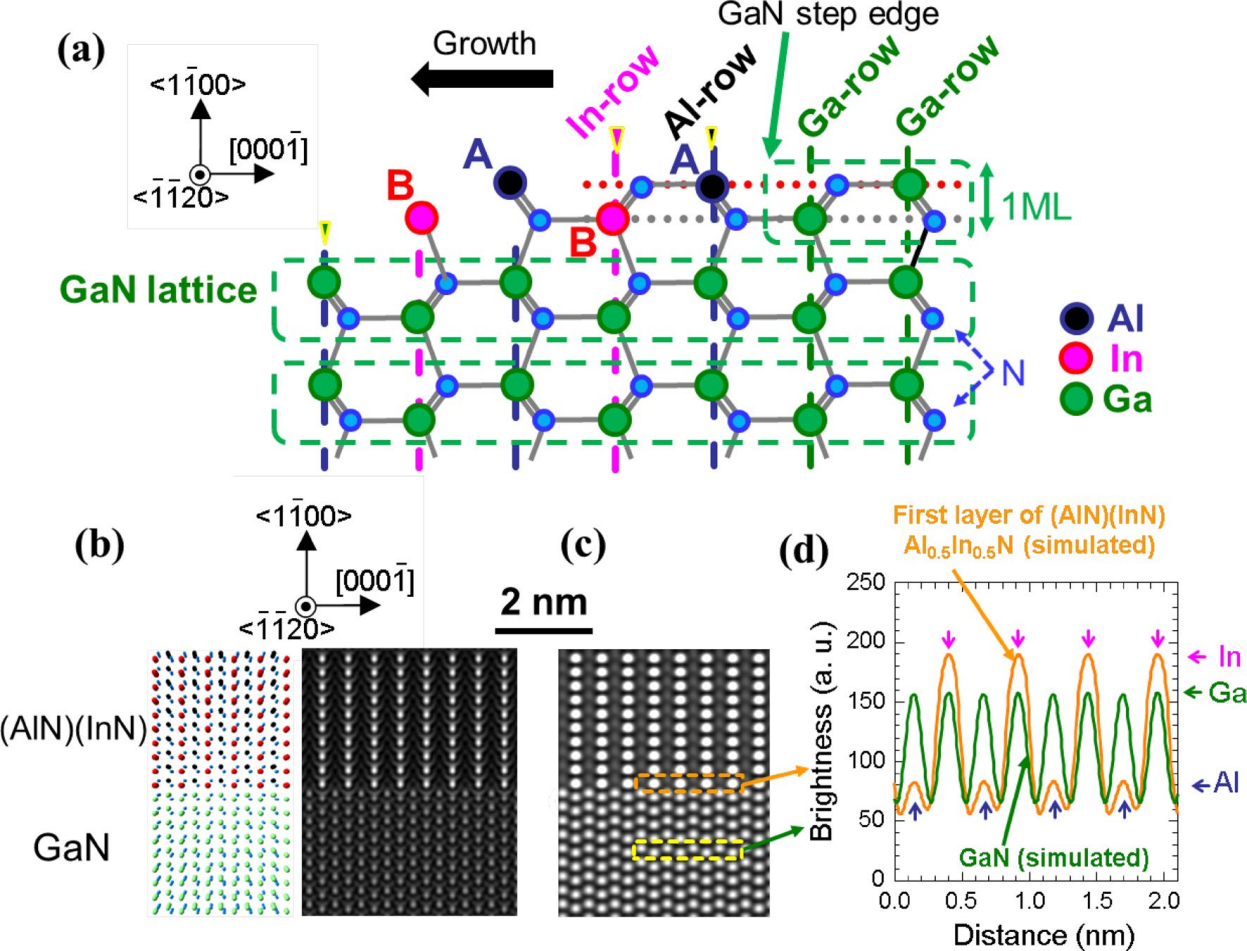
(**a**) A schematic drawing of the model striped ordering of <$\stackrel{-}{1}\stackrel{-}{1}20$> In- and Al-rows along the <0001> direction on the GaN surface. (**b**) A picture in perspective and theoretically simulated HAADF image for cation ordered *m*-plane (AlN)(InN) superlattices (Al_0.5_In_0.5_N film) on GaN. (**c**) Detuned and contrast adjusted HAADF image of (**b**) by adding finite beam defocus to reproduce the experimental brightness data of pure binary GaN (Fig. 4b). (**d**) The brightness profiles drawn for GaN and the first Al_0.5_In_0.5_N layer using the image in (**c**).

In order to elucidate the origin of {$10\stackrel{-}{1}2$} Al_0.74_In_0.26_N/Al_0.70_In_0.30_N CSLs, high-resolution cross-sectional HAADF-STEM images of the pseudomorphic Al_0.72_In_0.28_N/GaN heterointerface are shown in Figs. 4 and 5. There are two distinct length-scale orderings, as follows. In the HAADF image taken from (0001) cross-section, well-aligned hexagonal-shaped cation images are observed, as shown in Fig. 4a. Because cation and nitrogen atoms align on <0001>-axes, cation images are predominantly seen. Consistent with the magnitude of average *Z*, hexagons in GaN are brighter than those in Al_0.72_In_0.28_N. However, the brightest horizontal row is found at very Al_0.72_In_0.28_N/GaN interface, indicating the highest *x* at the first Al_1−*x*_In_*x*_N layer. This interface segregation of In is further evidenced by HAADF-STEM observation from {$11\stackrel{-}{2}0$} cross-section. As shown in Fig. 4b, brightest dots appear every 0.52 nm (two cation planes) at the first Al_1−*x*_In_*x*_N layer, which maintains ML atomic steps with terrace widths of approximately 70 nm (Fig. 3c). The results indicate that cation sublattices along <0001>-axis are alternately occupied by In atoms (or extremely In-rich Al_1−*x*_In_*x*_N) and Al atoms (or extremely Al-rich Al_1−*x*_In_*x*_N). At the second Al_1−*x*_In_*x*_N layer, the ordinality suddenly lessens but there still exists a 0.52-nm-period ordering: fin-shaped In-rich (bright) and In-poor (dim) (0001)-planes alternately appear every 0.52 nm along <0001> direction, as shown by magenta and dark blue triangles in Fig. 4b. For quantitatively observing such orderings, brightness profiles for this image enclosed by rectangles in Fig. 4b are shown in Fig. 4c–e. For underlying GaN, a 0.26-nm-period oscillating brightness profile is found in Fig. 4d. Its peaks correspond to the signals from Ga and valleys to unextinguished background signals. We note that the spatial resolution for our electron diffraction was insufficient to resolve closely located N signals from that of cations. Diffrent from GaN, brightness profiles for the first layer and the bulk of Al_1−*x*_In_*x*_N exhibit higher and lower, two-level peaks alternately with common valleys, as shown in Fig. 4e. The intensity ratio for the two levels in the first layer profile (top trace in Fig. 4e) is larger than that in the Al_0.72_In_0.28_N epilayer profile (bottom trace in Fig. 4e), indicating higher ordinality at the first layer. In Fig. 4c, a vertical brightness profile across the interface is displayerd. Although slight differences are recognized, cation signals in GaN, the first layer, and Al_0.72_In_0.28_N epilayer exhibit corresponding brightness features. We note that the brightness of the first layer cation is much higher than those in GaN or Al_0.72_In_0.28_N zones, indicating significantly large *Z*.

For explaining the formation dynamics of these orderings, the surface atomic structure near the step-edge is schematically drawn in Fig. 5a. Here, the step-flow growth appears to proceed toward [0001] direction, judging from the shape of the ML atomic step in Fig. 4b. The striped ordering of < $\stackrel{-}{1}\stackrel{-}{1}20$ > In- and Al-rows (or extremely In-rich Al_1-*x*_In_*x*_N-row and extremely Al-rich Al_1-*x*_In_*x*_N-row) on GaN along [0001]-axis is most likely formed by site-selection of cation species at the growth front, as follows. On each terrace of atomically-flat *m*-plane GaN, In and Al species diffuse toward the ML step-edge. Because large molar flow ratio (CH_3_)_3_In: (CH_3_)_3_Al = 8:1 was used to obtain^7^ solid-phase *x* of 0.3 (Ref.^32^), In species tend to cover the surface. However, uppermost and second uppermost < $\stackrel{-}{1}\stackrel{-}{1}20$ > cation-rows, which are indicated by **A** and **B** in Fig. 5a, are most probably occupied by Al and In, respectively. This site selection can be understood, as follows. Because the cohesive energies of AlN and InN, respectively, are − 5.83 and − 3.99 eV/atom (experimental)^47^ and − 5.853 and − 4.195 eV/atom (first-principles calculations)^48^, the configuration “two Al-N bonds at **A** and one In-N bond at **B**” is more stable than that of “one Al-N bond at **B** and two In-N bonds at **A**”. These adatoms are temporally pinned by N-bridges, and subsequent growth of the next involatile < $\stackrel{-}{1}\stackrel{-}{1}20$> Al-row (**A**) buries the volatile < $\stackrel{-}{1}\stackrel{-}{1}20$ > In-row (**B**) at the second from the top. Consistent with this model, < $\stackrel{-}{1}\stackrel{-}{1}20$> Al-rows on the In-poor (0001) planes in Fig. 4b are found at slightly below and more slightly above < $\stackrel{-}{1}\stackrel{-}{1}20$> In-rows.

In Fig. 5b, a picture in perspective and theoretically simulated HAADF image for a model *m*-plane, *c*-directional (AlN)(InN) superlattices (Al_0.5_In_0.5_N film) on GaN are shown to assess the perfection of Al/In ordering at the first layer. Figure 5c shows the detuned HAADF image of Fig. 5b by applying finite beam defocus to reproduce the experimental brightness profile of pure binary GaN (Fig. 4b). The brightness profiles drawn for GaN and the first Al_0.5_In_0.5_N layer of Fig. 5c are given in Fig. 5d. For Al_0.5_In_0.5_N, the ratio of the two peak levels is slightly larger than that in Fig. 4e, implying that the first layer is an essentially but not necessarily pure self-formed Al_0.5_In_0.5_N. At the second Al_0.72_In_0.28_N layer, the ordinality suddenly lessens in comparison with the first layer (Fig. 4b), because the influences of Ga bonds are not so significant at the second nearest neighbor cation site. However, In-rich/In-poor < $\stackrel{-}{1}\stackrel{-}{1}20$> rows are still alternately formed, which grow into respective {0001} planes resulting in their fin-shaped ordering along [0001]-direction (Fig. 4b).

## Discussion 2: {$10\stackrel{-}{1}2$} Al_0.74_In_0.26_N/Al_0.70_In_0.30_N CSLs

**Figure 6:**
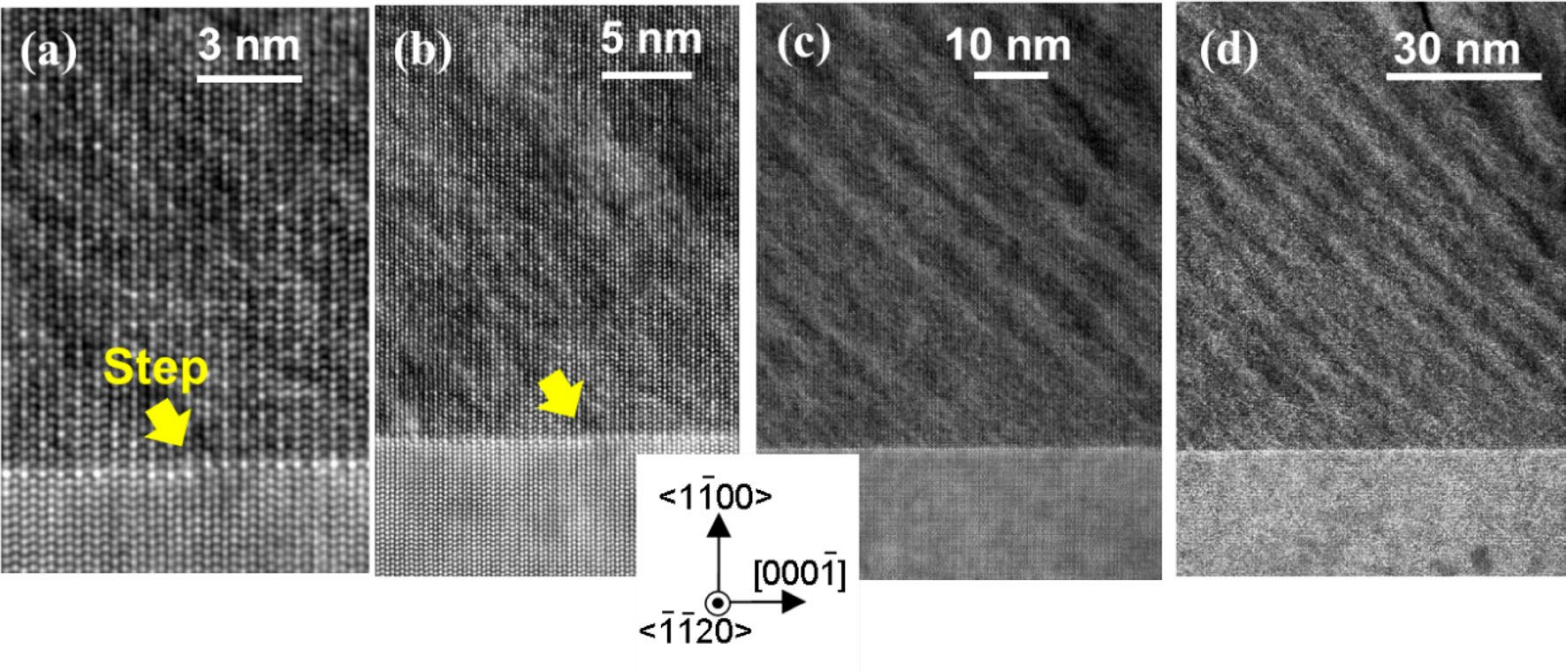
Cross-sectional HAADF-STEM images for the *m*-plane pseudomorphic Al_0.72_In_0.28_N/GaN heterointerface taken from the {$11\stackrel{-}{2}0$} cross-section. In panels (a) and (b), the location of the ML atomic step is indicated by the arrow. The step-flow growth proceeds toward the left side. The starting points of Al_0.74_In_0.26_ N (dim) and Al_0.70_In_0.30_N (bright) stripes parallel to the {$10\stackrel{-}{1}2\}$ plane are independent of the locations of surface ML steps.

Simultaneously to the lattice parameter scale ordering, the other length-scale ordering, namely approximately 5-nm-period {$10\stackrel{-}{1}2$} Al_0.74_In_0.26_N/Al_0.70_In_0.30_N CSLs, is initiated at the second Al_0.72_In_0.28_N layer on *m*-plane GaN: a few-nm-wide two-tone stripes, namely a few-nm-wide InN-rich (Al_0.70_In_0.30_N) and InN-poor (Al_0.74_In_0.26_N) < $\stackrel{-}{1}\stackrel{-}{1}20$> zones, are observed at the second Al_0.72_In_0.28_N layer, as shown in Figs. 4b and 6a–d. The locations of boundary-lines of these CSLs seem to be independent of step-edge locations, indicating that the Al_0.70_In_0.30_N/Al_0.74_In_0.26_N zones are most likely formed to mitigate gross lattice-mismatch between Al_0.72_In_0.28_N and GaN because the lattice mismatch (Δ*c*) between InN and GaN (11%) is much larger than that of AlN and GaN (3%) and hence InN-poor zones are periodically formed on the *m*-plane Al_0.5_In_0.5_N (or *x* close to 0.5) ML on GaN structure. We note that Al_0.74_In_0.26_N lattice-matches to GaN along <0001>-axis. Because step-flow growth progresses toward <0001> Ga-polar direction, a-few-nm-period Al_0.70_In_0.30_N/Al_0.74_In_0.26_N progresses toward upper left on {$10\stackrel{-}{1}2$} plane, which gives rise to the formation of approximately 5-nm-period Al_0.70_In_0.30_N/Al_0.74_In_0.26_N CSLs along the <$20\stackrel{-}{2}1$>-axis, as seen in Figs. 2c,d, 3c,f,g, 4b and 6.

## Discussion 3: lattice relaxation and generation of Al_0.68_In_0.32_N nanoboards

The pseudomorphic growth of CSLs lasts approximately 50 to 60 nm until the accumulated stress exceeds the elastic limit of the film. This value is close to what has been reported for *c*-plane lattice-matched Al_0.82_In_0.18_N film growth on GaN^21^. Then lattice relaxation takes place in Transition zone: the continuous film growth appears to terminate at the areas encircled in Fig. 3c,d. As most of the spatial gaps are initiated in the dim (In-poor) Al_0.74_In_0.26_N stripe, In-desorption or Al-precipitation may interfere the film growth. Once the gaps appear, the nanoboards having {$10\stackrel{-}{1}3$} facets start growing. The reason for the appearance of {$10\stackrel{-}{1}3$} facets can be understood from Fig. 5a that the lack of an In atom would stop growing {$10\stackrel{-}{1}2$} and skip to {$10\stackrel{-}{1}3$} facets. Above Transition zone, a random compositional inhomogeneity shown in Fig. 3a,b is introduced in the Al_0.68_In_0.32_N nanoboards.

## Discussion 4: correlation between the structural and optical properties

**Figure 7:**
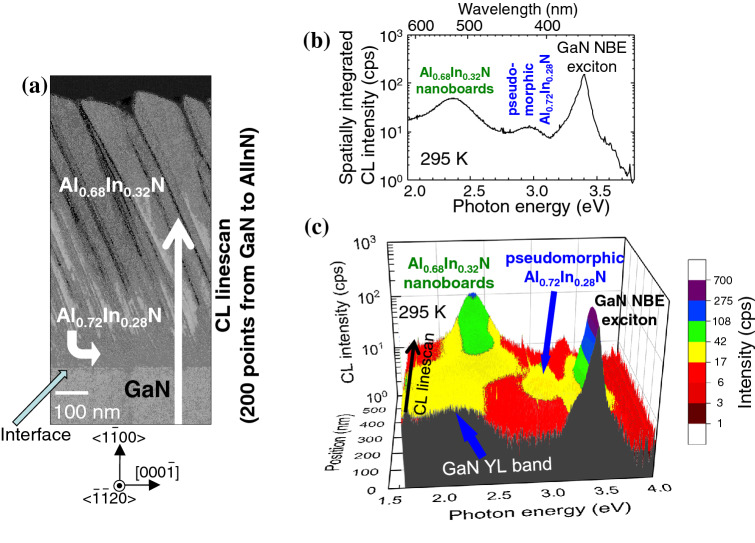
Spatially resolved CL spectra of the *m*-plane Al_0.7_In_0.3_N epitaxial structure. (**a**) Cross-sectional HAADF-STEM image of the *m*-plane Al_0.7_In_0.3_N epitaxial structure observed from {$11\stackrel{-}{2}0$} cross-section. (**b**) Spatially integrated cross-sectional CL spectra measured at 295 K for the area including GaN, Pseudomorphic zone, Transision zone, and nearly half of Nanoboards zone in (**a**). (**c**) Cross-sectional SRCL spectra measured at 200 points along the arrow labeled “CL linescan” in (**a**) using a 3D representation. The CL spectra in the *m*-plane GaN, pseudomorphic Al_0.72_In_0.28_N zone, and Al_0.68_In_0.32_N nanoboards are found from front to back of the figure. The intensities are shown by a contour map. Panel (a) is reproduced with permission from Chichibu et al.^23^.

Cross-sectional HAADF-STEM image and room temperature SRCL spectra of the *m*-plane Al_0.7_In_0.3_N/GaN epitaxial structure are compared in Fig. 7 to identiy the origins of particular CL peaks. The HAADF-STEM image shown in Fig. 2c is reloaded in Fig. 7a for easy comparison. Figure 7b shows the spatially integrated CL spectrum measured for the area including GaN, Pseudomorphic zone, Transision zone, and nearly half of Nanoboards zone in Fig. 7a. In Fig. 7c, cross-sectional SRCL spectra measured at 200 points along the arrow labeled “CL linescan” in Fig. 7a are shown using a 3D representation: CL spectra of the *m*-plane GaN, pseudomorphic Al_0.72_In_0.28_N zone (CSLs), and Al_0.68_In_0.32_N nanoboards are found at front to back of the figure. For GaN, an NBE emission originating from the recombination of free excitons (FX)^49^ at 3.41 eV dominates the spectra, while the intensity of the broad luminescence band at around 2.2 eV named “yellow luminescence (YL) band”^50–53^ is more than an order of magnitude weaker than the FX peak. Because YL band has two independent origins, namely carbon on nitrogen site (C_N_)^53^ and defect complex comprises of a Ga-vacancy (V_Ga_) and oxygen on nitrogen site (O_N_), V_Ga_O_N_^51^, the CL result indicates that our GaN homoepitaxial epilayer is of good purity with low C_N_ and V_Ga_ concentrations. As the focused electron beam (e-beam) position moves from GaN to the pseudomorphic Al_0.72_In_0.28_N zone, FX peak of GaN dissapears and a new peak at around 2.96 eV appears. According to the relationship between *E*_g_ and *x* in Al_1-*x*_In_*x*_N^20,23,28–30,32,34,41–46^, *E*_g_ of the In-rich stripe in the CSLs (Al_0.70_In_0.30_N) is estimated to be approximately 3.0 eV. Therefore, the peak at 2.96 eV is assigned as an NBE emission of the CSLs. We note that *E*_g_ of the In-poor counterpart (Al_0.74_In_0.26_N) is estimated from the *E*_g_-*x* relationship^20,23,28–30,32,34,41–46^ to be about 3.4 eV. Accordingly, SS of at least 0.4 eV will be produced in the present CSLs. The YL band of GaN is also observed when Pseudomorphic zone is excited. Because the diameter of an excitation volume using a 5 keV e-beam is about 100 nm, the result implies that some carriers excited in GaN are trapped by the origin of YL band^50–53^. In addition, the NBE emission of Pseudomorphic zone (2.96 eV) likely excites YL (2.2 eV) in the transparent GaN (*E*_g_ = 3.43 eV). Nevertheless, spot-excitation CL made it possible to detect the emission from the 50-nm-thick CSL layer. Finally, Al_0.68_In_0.32_N Nanoboards zone exhibits a predominant green emission band at around 2.36 eV, the energy agrees with the emission peak of our VFD device (Fig. 1). As *x* in Nanoboards zone distributes from 0.26 to 0.35, as shown in Fig. 3b, it is reasonable that the NBE emission in the nanoboards exhibits SS of about 0.7 eV^29,30,32^. As can be seen in Fig. 1, the emission from Nanoboards zone is sufficiently visible under the fluorescent lamp illumination. The use of the 50-nm-thick Pseudomorphic CSLs on GaN as a light-emitting medium is more attractive, since the CSLs contain low densities of TDs.

In conclusion, the appearance and self-formation sequence of 0.52-nm-period striped ordering of <$\stackrel{-}{1}\stackrel{-}{1}20$> In/Al (or extremely In- and Al-rich Al_1−*x*_In_*x*_N) cation-rows at the first layer on *m*-plane GaN and fin-shaped In-rich/In-poor {0001}-planes from the second Al_0.72_In_0.28_N layer, as well as approximately 5-nm-period {$10\stackrel{-}{1}2$} Al_0.70_In_0.30_N/Al_0.74_In_0.26_N CSLs in 50 to 60-nm-thick pseudomorphic *m*-plane Al_0.72_In_0.28_N film^23,32^ during MOVPE were exemplified. Then, so-called composition-pulling^21,40^ phenomenon was described from the viewpoints of surface kinetics and strain relaxation in lattice-mismatched heteroepitaxy for explaining the growth of strain-relaxed Al_0.68_In_0.32_N nanoboards^23^ with random compositional inhomogeneity. Finally, cross-sectional SRCL spectra were correlated with these particular zones to explain large SSs and short *L*_p_. The use of pseudomorphic CSLs as light-emitting media was proposed.

## Methods

### Samples

Approximately 700-nm-thick unintentionally-doped *m*-plane Al_0.7_In_0.3_N epilayer was grown^32^ subsequently to a 1000-nm-thick GaN homoepitaxial buffer layer by MOVPE on a 8 × 20-mm^2^-area, 325-μm-thick *m*-plane FS-GaN substrate^54^, which was grown by hydride vapor phase epitaxy using GaCl and NH_3_. The TD and basal-plane stacking fault densities of the GaN epilayer grown using (CH_3_)_3_ Ga and NH_3_ were lower than our detection limits (< 1 × 10^6^ cm^−2^ and < 1 × 10^3^ cm^−1^, respectively). The nominaly *x* = 0.3 (Al_0.7_In_0.3_N) epilayer was grown using (CH_3_)_3_Al, (CH_3_)_3_In, and NH_3_ at *T*_g_ = 660 °C. The reactor pressure was 2.7 × 10^4^ Pa. A large molar flow rate ratio of (CH_3_)_3_In: (CH_3_)_3_Al = 8:1 was used to obtain the solid phase *x* = 0.3. The structural properties such as crystal mosaics, surface morphologies and strains are given in Ref. 32. Residual concentrations of oxygen, [O], carbon, [C], and silicon, [Si], in Al_0.7_In_0.3_N were semi-quantitatively determined by secondary-ion mass spectrometry using an AlN (*x* = 0) reference due to the lack of an Al_0.7_In_0.3_N reference. Consequently, their concentrations are generally overestimated because the corresponding ion signals in In-containing matrix are stronger than true values. For example, [O], [C], and [Si] in Al_0.7_In_0.3_N estimated in this way were about 10^21^, 10^20^, and 10^19^ cm^−3^, respectively. However, these values are of course too high and unreliable. Because electron concentration, *n*, in the FS-GaN substrate is as high as 2 × 10^18^ cm^−3^, correct characterization of *n* and electron mobility in overgrown Al_0.7_In_0.3_ N layer was not possible. We therefore speculate *n* in the Al_0.7_In_0.3_N layer to be higher than 10^19^ cm^−3^.

### Vacuum fluorescent display (VFD) devices

The information of the green VFD comprising of Al_0.7_In_0.3_N nanoboards displayed in Fig. 1 can be found in Ref.^23^.

### Scanning transmission electron microscopy (STEM) and high-angle annular dark field (HAADF) images

Bright-field and dark-field STEM images were taken using JEM-ARM200F operated at *V*_acc_ = 200 kV. The InN mole fraction *x* was evaluated by using nanoprobe EDX measurements using the same STEM equipped with JED-2300T detector. The EDX data were analyzed using the software implemented in the microscope named *NSS* Ver. 3.3.113 (Thermo Fisher Scientific), and chemical compositions were estimated using the Cliff-Lorimer method without standard samples. The overall error in the InN mole fraction *x* excluding the effects of x-ray absorption in our Al_0.7_In_0.3_N film was approximately $\pm$ 0.03. For example, *x* will be expressed as 0.30 $\pm$ 0.03. By using the same STEM, HAADF images were taken to observe slight changes in crystallographic orientation and/or chemical composition. For quantitative analysis of HAADF images, ideal images were simulated using the software named xHREM (HREM Research, Inc.)^55^. After calculating the image created for tightly focused e-beam with nearly zero broadening, the contrast was adjusted by adding finite beam defocus to reproduce experimental brightness profile of pure binary GaN.

### Spatially resolved cathodoluminescence (SRCL) measurement

Cross-sectional SRCL measurement was carried out to record local CL spectra using a detection system composed of a 14-cm-focal-length grating spectrometer (MicroHR) and a 1024 × 256 thermoelectrically-cooled open-electrode charge-coupled device (CCD) array, which are equipped on a scanning electron microscopy (JSM-880 modified)^5,56^. Designated line-scanning and spatially integrated CL spectra were measured using the external beam-scanning module. The *V*_acc_ and current of the e-beam were 5 kV and 500 pA, respectively. The corresponding excited carrier concentration is calculated to be lower than 10^15^ cm^−3^ when the average carrier lifetime is 30 ps. All SRCL measurements were carried out at 295 K.

## Data Availability

The data that support the findings of this study are available in this manuscript.
